# Synthesis of novel cross-linked s-triazine-containing poly(aryl ether)s nanoparticles for biological fluorescent labeling

**DOI:** 10.1080/15685551.2017.1281786

**Published:** 2017-02-02

**Authors:** Kun Zhang, Kai Yang, Shiyun Ai, Jing Xu

**Affiliations:** ^a^ College of Chemistry and Material Science, Shandong Agriculture University, Tai’an, P.R. China; ^b^ College of Resources and Environment, Shandong Agriculture University, Tai’an, P.R. China

**Keywords:** S-triazine, poly (aryl ether)s, nanoparticle, fluorescent labeling

## Abstract

A blue-fluorescence 2,4,6-tris(4-fluorophenyl)-1,3,5-triazine (TFPT) monomer was synthesized with high yield and high purity under mild reaction conditions. The TFPT, which had three active fluoric groups, was facilely incorporated into stable cross-linked fluorescent polymeric nanoparticles (FCPNs) via precipitation polymerization with 6-(4-hydroxyphenyl)pyridazin-3(2H)-one (HPZ). The FCPNs were highly dispersible in water and showed uniform size, intense blue fluorescence, and excellent biocompatibility, making them promising for live cell imaging label applications. This work has the potential to promote the exploitation of novel s-triazine monomers, and to provide a new view of functional crossing-linked polymers based on such monomers.

## 1. Introduction

Advances in nanotechnology techniques for diagnostics have created new avenues for bioimaging, drug delivery, bioanalysis, and other biomedical applications, because only a small sample volume is required [1–4]. Fluorescent labeling technologies have been applied widely in bioimaging, biolabeling, biodetection, immunoassays, and other biomedical applications [5–7]. Traditional fluorescent materials such as organic fluorescent dyes and quantum dots (QDs) have attracted much attention [8,9]. However, quantum dots are technically challenging to synthesize and not easily bioconjugated; moreover, significant concerns remain regarding their long-term toxicity *in vivo*. Commercial dye-impregnated microspheres tend to be quite large, making the cellular uptake of such particles more difficult.

In addition to organic dyes and QDs, which have been extensively studied, fluorescent conjugated polymer nanoparticles (CPNs) have recently emerged as an alternative class of fluorescent nanoparticles that show promise for use as probes in biological imaging. In particular, they are promising for such applications because they are nontoxic, biodegradable, biocompatible, uniform in size, highly stable, and because they display desirable functionalities such as a stable and bright emission [10–12]. However, one important issue in the preparation of CPNs is the aggregated state of the hydrophobic fluorophores in the CPN cores, which causes quenching of the fluorescence. Cross-linked polymeric nanoparticles that emit intense fluorescence in the aggregated state were reported recently; it was demonstrated that these particles showed intense fluorescence in an aqueous solution [13,14]. However, the cross-linking strategy is rarely applied and the biocompatibility of the cross-linked CPNs is inadequate. Thus, the development of robust synthetic routes for the preparation of novel biocompatible cross-linked fluorescent polymeric nanoparticles (FCPNs) is of great scientific interest.

S-triazine units have attracted great interest in the field of functional polymer materials synthesis because of their unique functionalities, which include high thermal stability, high electron deficiency, spatial coplanarity, and luminosity; these properties derive from the structural symmetry, and the presence of п-conjugated systems [15–17]. The synthesis some s-triazine derivatives is therefore of interest [18,19].

**Scheme 1.**
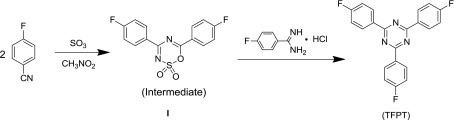
Synthesis route for TFPT.

The use of triazine-based cross-linked polymers for the fluorescent labeling of bacteria has not yet been explored. The introduction of these stable and fluorescent triazine units into an FCPN system could be advantageous for stability, as well as the manipulation of the fluorescent state of the polymers. In this context, we report for the first time the synthesis and characterization of cross-linked s-triazine-containing poly(aryl ether)s nanoparticles, and demonstrate their application as an optical indicator for the fluorescent labeling of *E. coli* bacteria (which were used as a model system). The key factor for the successful synthesis of the cross-linked s-triazine containing poly(aryl ether)s nanoparticles was obtaining a pure and high-yield s-triazine monomer. 2,4,6-tris(4-fluorophenyl)-1,3,5-triazine (TFPT) is a typical aromatic s-triazine monomer that has three active fluoric groups that can react with phenolic hydroxyl groups via nucleophilic substitution reactions for cross-linking poly (aryl ether)s [17,20–22]. To date, little attention has been given to this monomer. On the basis of existing literature data and the relevant theory, we ascertained that the major factors holding back progress with this monomer are the low yields and high costs associated with existing synthesis methods. Jürg Hulliger’s team reported the ZnCl_2_-catalyzed polymerization of 4-fluorobenzonitrile (performed for 64 h at 270 °C) to form TFPT, which was applied in the formation of inclusions featuring channels [23]. However, the yield (40%) was low for such a long reaction time (64 h) and high temperature (270 °C). Few studies of TFPT-based polymers have been reported, despite that fact that TFPT can react efficiently with phenolic hydroxyl groups. To address these issues, we first synthesized TFPT with a high yield, using Shiceru Matsuo’s method with some modifications (Scheme 1 **)** [21].

**Scheme 2.**
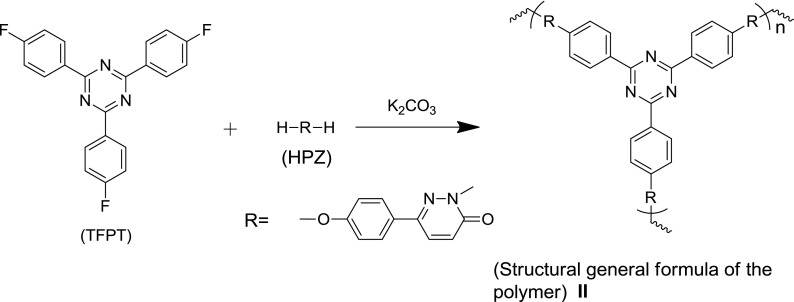
Synthesis route for cross-linked s-triazine-containing poly (aryl ether).

**Scheme 3.**
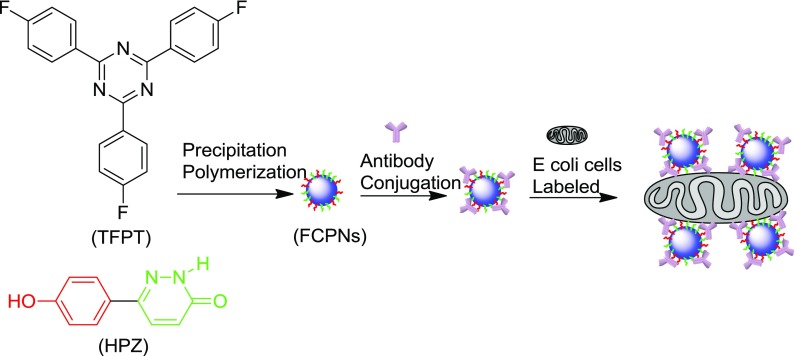
Schematic illustrating the preparation of the s-triazine-containing FCPNs.

The fluorescent cross-linked polymer nanoparticles (FCPNs) were prepared using TFPT and 6-(4-hydroxyphenyl) pyridazin-3(2H)-one (HPZ), via precipitation polymerization (Schemes 2 and 3). The morphology, thermal stability, fluorescent properties, and antibacterial activity of the nanoparticles were investigated in detail. We also demonstrated the practical application of these nanoparticles for the fluorescent labeling of live *E. coli*.

## 2. Experimental

### 2.1. Chemicals and reagents

6-(4-hydroxyphenyl)pyridazin-3(2H)-one (HPZ) was synthesized according to a procedure reported in the literature [24]. The monomer was a yellow powder. Yield: 80%. Purity: 100% [high-performance liquid chromatography (HPLC)]. mp: >300 °C. ^1^H NMR (400MHZ, DMSO-*d*_*6*_, ppm): 6.83, 7.67 (d, 2H), 6.90, 7.90 (d, 1H), 9.87 (s, 1H), 12.96 (s, 1H). Anal. calcd for C_10_H_8_N_2_O_2_: C 63.82, H 4.28, N 14.89; found: C 63.52, H 4.35, N 14.78.

4-Fluorobenzamidine-hydrochloride (Yiji, China), 4-fluorobenzonitrile (Aladdin, China), and sulfur trioxide (Spectrum China, China) were used as received. Tris–HCl buffer (pH 7.4) was prepared by mixing 50 mL of 0.1 mol/L tris(hydroxymethyl) aminomethane with 42 mL of 0.1 mol/L of hydrochloric acid. *E. coli* (K12 strain) was provided by the Collage of Life Sciences in Shandong Agricultural University. Rabbit Anti-*E. coli* DH5α polyclonal antibody was purchased from Beijing Hapten and Protein Biomedical Institute, China. All other solvents were of analytical grade, and were used without further purification.

### 2.2. Measurements

Infrared measurements were performed on a Thermo Nicolet Nexus 470 Fourier transform infrared (FTIR) spectrometer, at a resolution of 4 cm^−1^, using Nicolet 380 (Nicolet, US). ^1^H NMR and ^13^C NMR spectra were recorded on a Bruker 400 M spectrometer at an operating temperature of 25 °C, using H_2_SO_4_-d_2_ as a solvent, and were listed in parts per million downfield from tetramethylsilane (TMS). Elemental analyses were carried out using a Perkin Elmer model 2400 CHN analyzer, operated at 975 °C under nitrogen. The analytical HPLC method applied for the organic compounds was performed using a Shimadzu analysis chromatography system equipped with a Shimadzu SPD-M20A diode array detector (Shimadzu, Kyoto, Japan). An HPLC method was developed using a reversed-phase C18 column (Shiseido PAK-UG-120-61504, 250 mm × 4.6 mm I.D., 5 μm). The sample injection volume was 20 μL, and the column temperature was 30 °C. The mobile phase was 100% methanol. The flow rate was maintained at 1.0 mL/min, and the fluorescence detector was operated at 271 nm. The glass-transition temperature (*T*_g_) was determined using differential scanning calorimetry (DSC) (TAQ10, US), which was performed in a nitrogen atmosphere, from 50 to 350 °C, using a scan rate of 10 °C/min. The fluorescence of the samples was studied using an RF-5301PC spectrophotometer. All characterization measurements were performed at room temperature. The surface profile of the nanoparticles was investigated using a JEOL JEM-1400 TEM operating at 120 kV, and images were obtained as digital micrographs using a Gatan Multiscan CCD. To prepare samples for TEM characterization, the prepared nanoparticles were dispersed in water and cast on a copper grid. Fluorescent *E. coli* labeling images were obtained using an Olympus BX51 fluorescence microscope with an Olympus DP72 camera.

### 2.3. Synthesis of 2,4,6-tris(4-fluorophenyl)-1,3,5-triazine (TFPT)

The intermediate 4,6-bis(4-fluorophenyl)-1,2,3,5-oxathiadiazine 2,2-dioxide **I** was synthesized according to Shiceru Matsuo’s method, with a small modification [21]. 28.46 g (0.235 mol) of 4-fluorobenzonitrile was dissolved in 60 mL of nitromethane, and then cooled to 10 °C under constant stirring. Approximately 10.0 g of sulfur trioxide was added dropwise to the reaction mixture that should be kept below 50 °C. After an additional 30 min of stirring at room temperature, the solution was poured into diethyl ether (1:2 by volume) to precipitate the product. The product was filtered, washed with diethyl ether and methanol, and then dried to give 26.48 g of the intermediate **I**, with a 70% yield.

5.28 g (0.0164 mol) of intermediate product **I**, 3.43 g (0.0197 mol) of 4-fluorobenzamidine-hydrochloride, and 80 mL of acetone were added to a flask. 5.08 g (0.0453 mol) of potassium tert-butoxide was added slowly to the mixture, under vigorous stirring at room temperature, over a period of 30 min. After an additional 30 min of stirring at 50 °C, the mixture was poured into 300 mL of water. The needle-like solid TFPT was filtered, washed with methanol, and then dried at reduced pressure. The product was obtained as white needle-shaped crystals. Yield: 85% (5.07 g); Purity: 100% (HPLC); mp: 261.2–262.3 °C. FTIR (KBr, cm^−1^): 3061 (w, =C–H), 1600, 1523, 1507 (s, C=C, C=N), 1414 (m, C–N), 859, 816 (m, γ=C–H), ^1^H NMR (400 MHz, CDCl_3_, ppm) *δ*: 8.86–8.69 (m, 6H), 7.40–7.17 (m, 6H). Anal. calcd for C_21_H_12_N_3_F_3_: C 69.42, H 3.33, N 11.57; found: C 68.90, H 3.36, N 11.46.

### 2.4. Precipitation polymerization

A 500 mL-capacity, two-necked round-bottom flask equipped with a magnetic stirrer, dry nitrogen inlet and outlet, and a condenser was flushed with nitrogen, and then charged with HPZ (0.2632 g, 1.4 mmol), TFPT (0.3402 g, 0.93 mmol), K_2_CO_3_ (0.2512 g, 1.82 mmol), N-methyl-2-pyrrolidone (NMP) (210 mL), and toluene (250 mL). The water formed during the reaction was removed using a Dean–Stark trap for 1 h at 150 °C. The toluene was removed entirely when the reaction temperature was raised to 168 °C, and the sample was subjected to light stirring for 5 h. After the reaction sample had cooled to room temperature and had been left overnight, the nanoparticles **II** were collected, and then washed and centrifuged successively with N,N-dimethylacetamide (DMAC) and water. The product was dried at 70 °C in vacuo for 12 h. Yield: 37% (0.2232 g). FTIR: 3446 cm^−1^ (O–H, N–H), 1679 cm^−1^ (C=O), 1596 cm^−1^, 1499 cm^−1^, 1412 cm^−1^ (C=N, C=C), 1260 cm^−1^ (C–O–C), 3060 cm^−1^, 874 cm^−1^, 819 cm^−1^ (C–H), Solid ^13^C NMR (400 MHz, H_2_SO_4_-*d*_*2*_, ppm): 222.5, 214.0, 199.2, 180.7, 170.5, 163.7, 159.1, 144.3, 130.1, 121.2, 80.1, 34.7. Anal. calcd for C_51_H_30_N_9_O_6_: C 70.58, H 3.83, N 14.53; found: C 71.02, H 3.85, N 14.37.

### 2.5. Biocompatibility tests

To assess the biocompatibility of the nanoparticles, the *E. coli* suspension was diluted in a nutrient broth to approximately 103–104 colony-forming units (CFU) in 1 mL. The as-prepared sample (10 mg of nanoparticles dispersed in 900 μL of nutrient broth) was inoculated with 100 μL of the bacterial suspension; the blank control was diluted to the same concentration. After the bacteria-inoculated solutions were incubated in a shaking incubator at 37 °C for 1 h, 50 μL of each solution was taken and cultured on Luria-Bertani (LB) agar plates and maintained at 37 °C for 24 h in a standing-temperature cultivator. The bacterial colonies were then observed.

### 2.6. Fluorescent labeling of E. coli

0.1 mg of the nanoparticles was dispersed in 1 mL of a Tris–HCl buffer solution, and reacted with rabbit anti-*E. coli* DH5α polyclonal antibody (100μL) at room temperature for 3 h. 100 μL of the *E. coli* suspension (105–106 CFU/mL) was then incubated in the mixture, under shaking, at 37 °C for 1 h. The suspension was then examined using fluorescence microscopy.

## 3. Results and discussion

### 3.1. Monomer synthesis and characterization

As shown in Scheme 1, 2,4,6-tris(4-fluorophenyl)-1,3,5-triazine (TFPT) was synthesized using 4-fluorobenzamidine-hydrochloride and 4,6-bis(4-fluorophenyl)-1,2,3,5-oxathiadiazine-2,2-dioxide (heterocycle **I**), based on the reaction of 4-fuorobenzonitrile with SO_3_ in nitromethane. To obtain the TFPT monomer with a high yield and high purity, certain experimental considerations were important. First, the appropriate dosage of sulfur trioxide had to be applied, using a molar ratio of nitrile:SO_3_ of 2:1. Greater or lesser amounts of sulfur trioxide decreased the yield and purity of heterocycle **I**. Second, the sulfur trioxide had to be introduced slowly, and the reaction temperature had to be maintained at less than 50 °C, to avoid any sulfonation reaction or TFPT formation due to the self-reaction of heterocycle **I**. If these conditions were not applied, only a low yield (2%) of TFPT was obtained. If the required conditions were applied, the crude product **I** (70% yield) could be used for the subsequent reaction directly, without further purification.

It has been shown that on heating with nitriles in the presence of HCl, 4,6-disubstituted 1,2,3,5-oxathiadiazine-2,2-dioxides are easily converted to triazines [25]. However, the self-cycloaddition of heterocycle **I** tends to occur alongside the above reaction, resulting in low yields of TFPT. To overcome this issue, several methods were adopted. It was found that acetone was a suitable relatively-low-polarity solution that could reduce the rate of the self-reaction and accelerate the reaction of heterocycle **I** with the 4-fluorobenzamidine-hydrochloride. In contrast, N, N-dimethylformamide was a negative example that produced a 5% yield of self-reaction products. Potassium tert-butoxide was chosen as the strong base to neutralize the generated sulfur trioxide and hydrogen chloride, and to facilitate the main reaction. The above methods reduced losses, and simplified the previously complex synthetic steps. Remarkably, the yield of TFPT was increased to 85% from synthesis performed at 50 °C for 1 h, and the purity of the TFPT produced was 100%, with no need for recrystallization. The monomer was identified using Fourier transform infrared (FTIR) analysis (Supporting Information Figure S1a). The characteristic C=N, C=C, and C–N stretching vibrations of the s-triazine rings were shown around 1600, 1523, 1507, and 1414 cm^−1^, respectively. The ^1^H NMR spectra for TFPT (shown in Figure S2) was easily recognized, as a result of the symmetrical structure, and the two peaks at approximately 8.75 and 7.3 ppm that indicated the presence of the only two kinds of hydrogen protons in the benzene rings.

### 3.2. Polymer synthesis and characterization

We reported previously 6-(4-hydroxyphenyl)-3(2H)-pyridazinone (HPZ), a biphenol-like monomer [24,26], which give poly(aryl ether)s in a nucleophilic aromatic substitution reaction with bifluorophenyl compound. Here, HPZ was reacted with TFPT in a precipitation polymerization (Scheme 2). To create the desired crossing-linked poly (aryl ether) nanoparticles, strongly polar aprotic NMP was chosen as the solvent [27]. The thus-obtained FCPNs could be separated using centrifugation, and then dispersed again in NMP or aqueous solutions using ultrasonication; this was of benefit for the further purification, application, and cycle collection. The structure was characterized using FTIR and solid ^13^C NMR (Supporting Information Figure S1b, S3). As shown in Figure S1b, peaks characteristic of TFPT were observed to be centered at 1600, 1522, and 1506 cm^−1^; these peaks were assigned to the stretching vibrations of the C=C and C=N bonds in the polymers. A peak characteristic of HPZ was located at 1680 cm^−1^, suggesting that C=O bonds were introduced into the polymers; the broad peak at approximately 3400 cm^−1^ indicated that the polymer was mostly end-capped with –OH or –NH groups. However, the connection pattern between TFPT and HPZ was complicated because of the various possible linkage sites on the HPZ moieties. The cross-linked polymer structure is shown as a general formula (**II**) in Scheme 2. Due to the various bonding ways between HPZ and TFPT in the cross-linked structure, as shown in Scheme S1 and Figure S3, the ^13^C NMR spectrum was too complex for a detailed structure. Nevertheless, the general structure of the cross-linked polymers could be verified, and the characteristic peak that was shifted downfield by 220–200 ppm was identified as being associated with the C=O groups of HPZ. The signals at 180 and 170 ppm were assigned to the carbon protons of the s-triazine ring. The sharp peak at approximately 130 ppm was assigned to the carbon protons of the benzene ring. It should be noted that a carbon resonance was detected at 163 ppm, further suggesting that phenolic hydroxyl groups end-capped the FCPNs.

**Figure 1.**
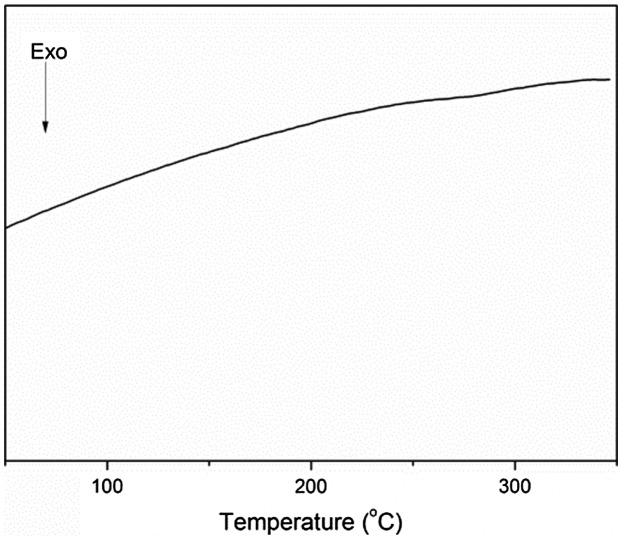
DSC curves for the polymer.

The transition temperature of the polymer was determined using DSC. As shown in Figure 1, the glass transition temperature of the polymer was not detected in the range of 50–350 °C, which indicated the high degree of cross-linking in the polymer structure. The slight fluctuation observed in the 270–290 °C range might have been owing to the amorphous structure of the end-capped HPZ moieties.

**Figure 2.**
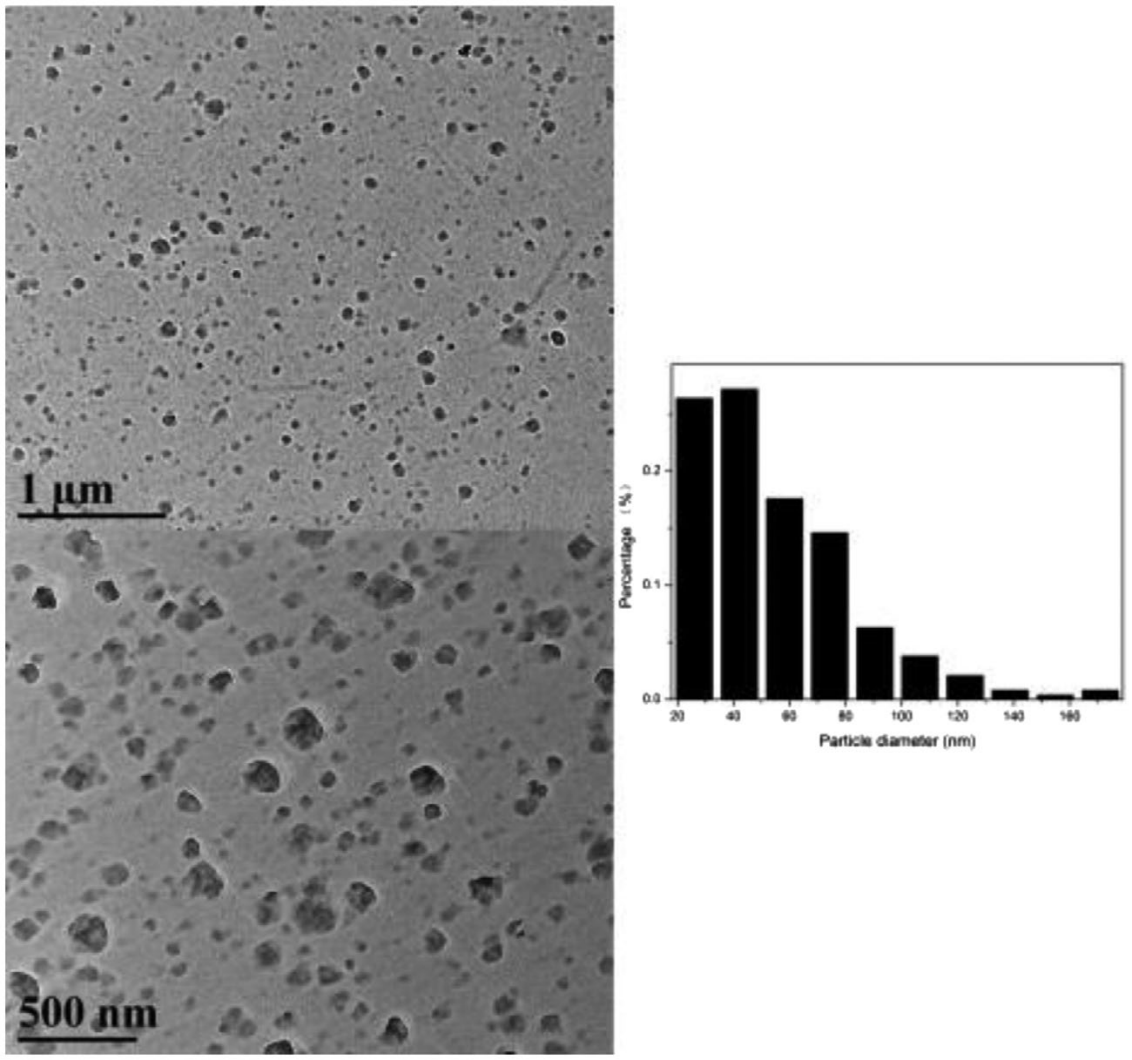
TEM images of the nanoparticles, and the particle size distribution.

Owing to the formation of hydrophobic aromatic groups as hydrophobic cores, –OH and –NH groups formed the shell surface, and the obtained cross-linked copolymers therefore showed amphiphilic properties. When dispersed in an aqueous solution, the hydrophilic group-covered surface tended to form hydrogen bonds with the water, resulting in a stable and highly dispersed system. Figure 2 shows the morphology of the cross-linked polymers, which was investigated using transmission electron microscopy (TEM). Many divided spheroidic nanoparticles with diameters ranging from 20 to 100 nm could be clearly identified.

**Figure 3.**
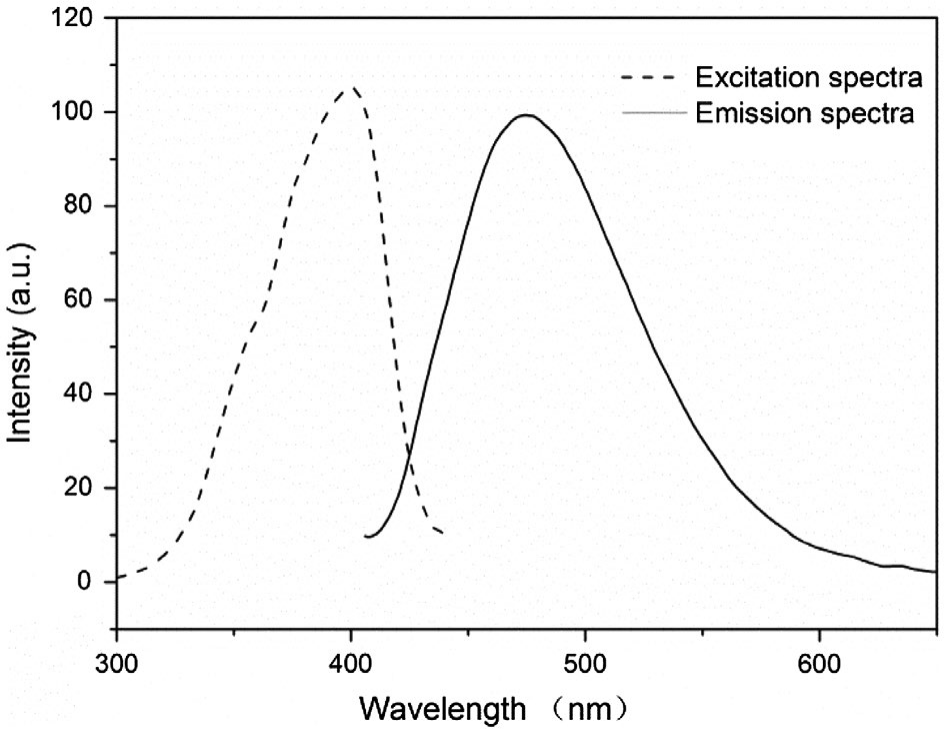
Fluorescent excitation and emission spectra for the nanoparticles.

**Figure 4.**
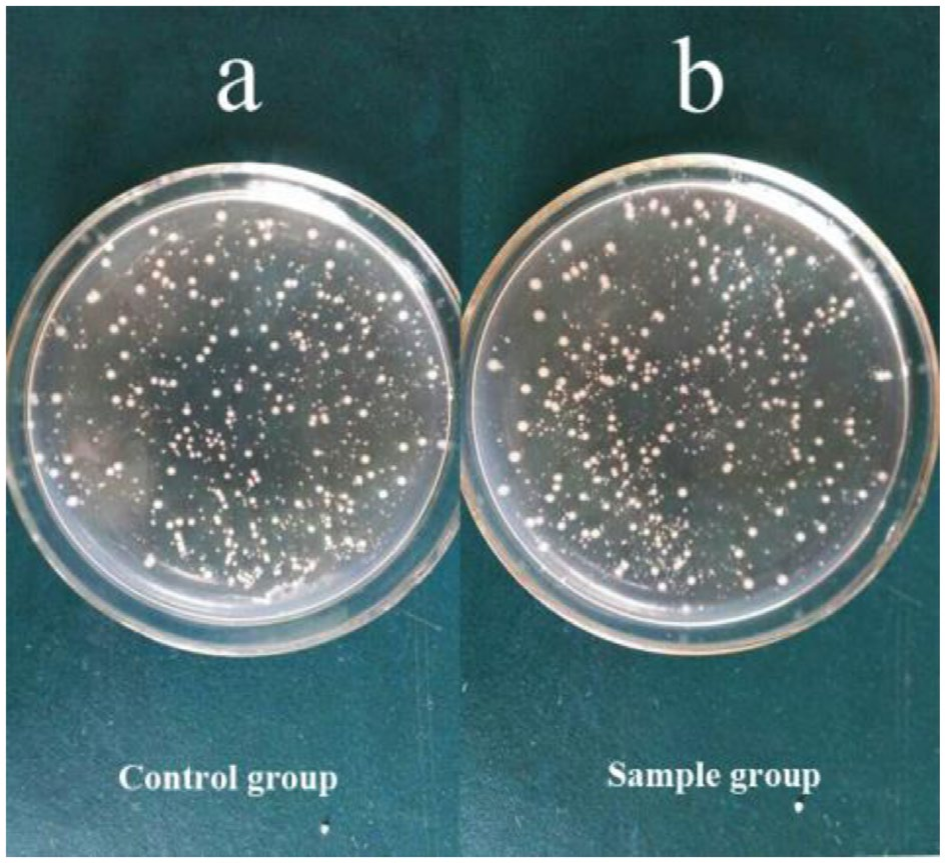
Biocompatibility evaluation of the s-triazine-containing FCPNs. Images of *E. coli* cells incubated with different concentrations of FCPNs at 37 °C for 24 h: (a) control cells, (b) 10 mg/mL.

**Figure 5.**
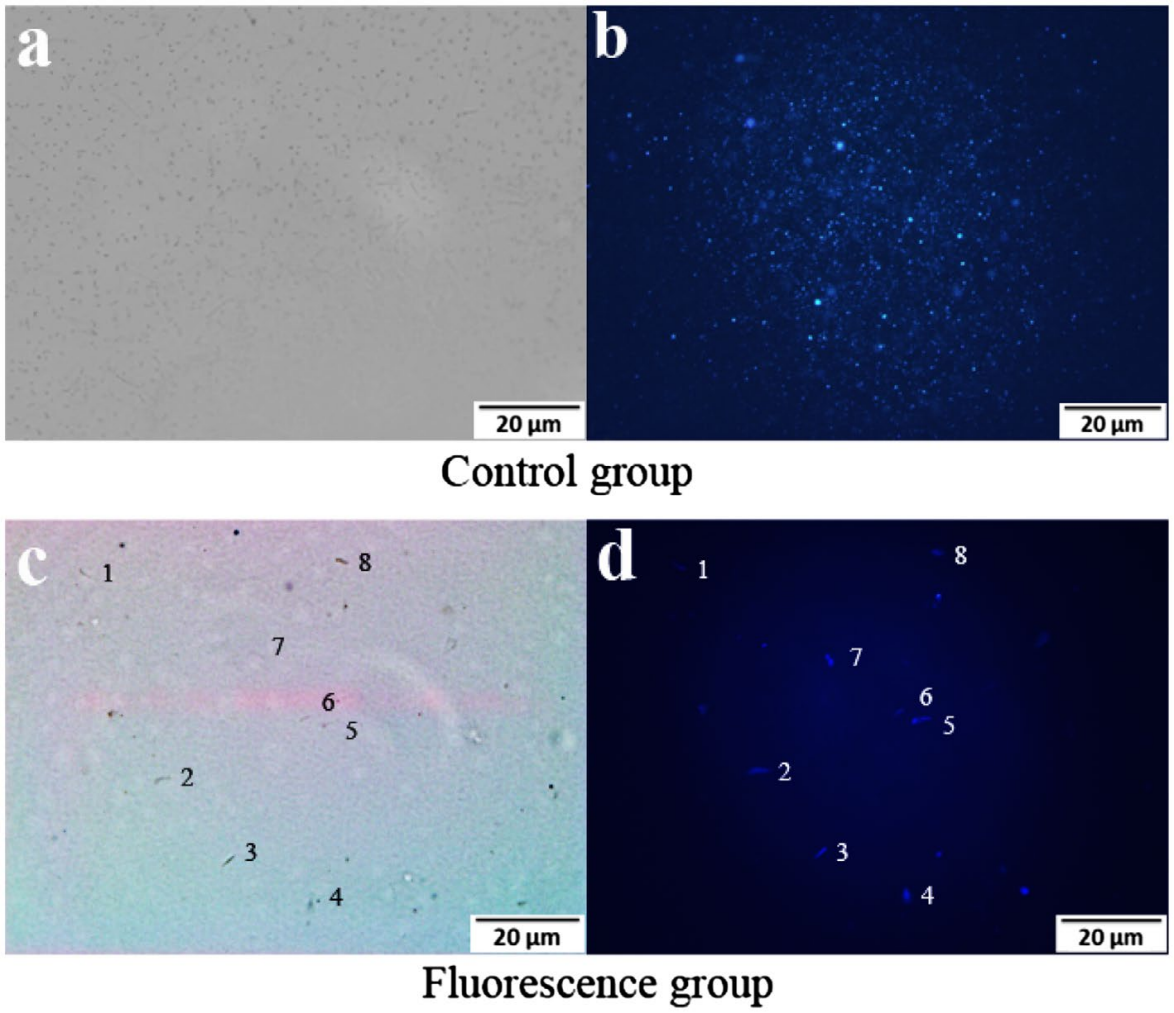
Fluorescence microscopy images of suspended nanoparticles (a, b), and live *E. coli* (c, d).

As illustrated in Scheme 1, the steric hindrance produced by the aromatic rings’ twisted and cross-linking structure was responsible for the polymer conjugate’s rigid plane structure. The s-triazine, and other hydrophobic groups were aggregated in the nanoparticle cores, and the obtained FCPNs were expected to show strong fluorescence, and to be highly dispersible in aqueous environments. As shown in Figure 3, the fluorescence emission spectrum measured for the FCPNs dispersed in water displayed a maximum emission peak at 481 nm, while the fluorescence excitation spectrum measured in water showed a strong excitation wavelength at 400 nm. The emission spectrum ascribed to the s-triazine unit was reported in studies investigating the synthesis of blue luminous materials [15,28]. The pyridazinone unit [29] and fluorophore =C=O act as the pivot of the conjugated structure, and the electron-donating groups (–OH, =NH) in the polymer terminal extend the conjugated system. These excellent fluorescent features are of great benefit for potential cell imaging labels applications.

### 3.3. Biocompatibility and potential application of FCPNs as live bioimaging labels

As mentioned earlier, the biocompatibility of fluorescent nanoparticles is a critical issue for their use in fluorescence organism imaging. Considering that the HPZ unit was widely used in the synthesis of pesticides, it was necessary to assess the cytotoxicity of the nanoparticles [12,29,30]. In this study, a biocompatibility assessment was carried out to evaluate the potential of the s-triazine and HPZ containing FCPNs for biomedical applications; this was achieved by measuring the viability of bacteria *E. coli* after the cells were incubated with different concentrations of FCPNs for 24 h. Figure 4 shows that the cell viability of the *E. coli* cells was close to 100%, even when the particle concentration was as high as 10 mg/mL; this demonstrated that the synthesized FCPNs had negligible cytotoxicity, and that the FCPNs show great potential for bioimaging applications. Furthermore, the biocompatible surface of the FCPNs could connect with *E. coli* antibodies via weak intermolecular forces and hydrogen bonds, because of the biocompatible –OH and –NH groups covering the surface of the nanoparticles. It is reasonable to give a viable option of application in fluorescence labeled bacteria by FCPNs linking with specific antibody [31–34].

To evaluate the performance of the s-triazine-containing FCPNs, and the living cells after labeling with the FCPNs, fluorescence microscopy images were taken for a 0.1 mg/mL nanoparticle suspension that reacted with Rabbit Anti-*E. coli* DH5α Polyclonal Antibody, and 10^4^–10^5^ CFU of *E. coli* in a Tris–HCl buffer solution. Bright-field images (Figure 5(a), (c)) of the control and fluorescence group were taken immediately prior to the capturing of the fluorescence microscopy images (Figure 5(b), (d)). As shown in Figure 5(a) and (b), the nanoparticles were highly dispersed in the aqueous solution, and appeared as bright blue spots. As shown in Figure 5(d), the labeled *E. coli* bacterial cells could be observed clearly as blue rod-like shapes in the dark field; they appeared as dark rod-like shapes in the bright-field image in Figure 5(c). The *E. coli* bacteria were successfully labeled with the FCPNs. Considering the lack of any cytotoxic effects on living cells, the targeting of these s-triazine-containing nanoparticles for drug delivery and imaging label applications will be of particular interest.

## 4. Conclusion

The fluorescent monomer 2, 4, 6-tris(4-fluorophenyl)-1, 3, 5-triazine (TFPT) was synthesized with high yield and high purity, using a simple two-step method. Biocompatible and blue-luminescent monodisperse nanoparticles were synthesized using a facile, one-pot precipitation polymerization of TFPT and 6-(4-hydroxyphenyl)pyridazin-3(2H)-one (HPZ). Biocompatibility and bioimaging tests were performed on the s-triazine-containing nanospheres by culturing them with living *E. coli* bacteria. The nanoparticles exhibited no cytotoxic effects, indicating these novel biocompatible cross-linked polymer nanoparticles as promising fluorescent platforms for live cell imaging applications. This work provides a platform for the future preparation of cross-linked, TFPT-based polymers, and for the exploitation of new applications in the field of cross-linked functional polymer synthesis.

## Disclosure statement

No potential conflict of interest was reported by the authors.

## Funding

This work was supported by the National Natural Science Foundation of China [grant number 31000938]; the Project of Shandong Province Education Department [grant number ZR2014JL023]; and the National Key Scientific Research Project [grant number 2016YFB0302400].

## Supplemental data

The supplementary material for this paper is available online at http://dx.doi.org/10.1080/15685551.2017.1281786.

## Supplementary Material

TDMP_1281786_Supplementary_Material.pdf
