# Supplementary material for: Synthesis of novel cross-linked s-triazine-containing poly(aryl ether)s nanoparticles for biological fluorescent labeling
Source: Des Monomers Polym. 2017 Feb 2;20(1):389–96. doi: 10.1080/15685551.2017.1281786 (PMC5784864; doi:10.1080/15685551.2017.1281786)
Supplement: TDMP_1281786_Supplementary_Material.pdf [file TDMP_A_1281786_SM5444.pdf]

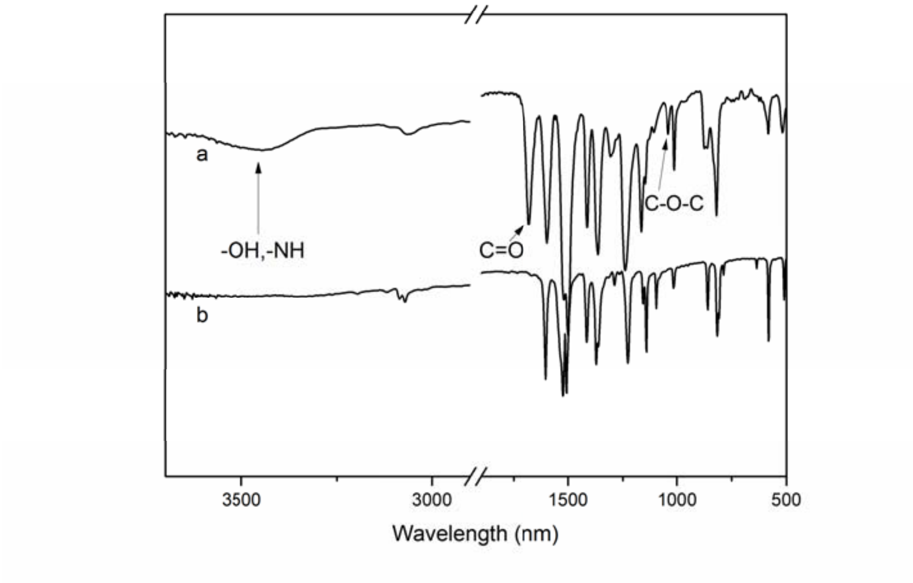

Figure S1.FTIR spectrafor polymer (a), and TFPT (b).

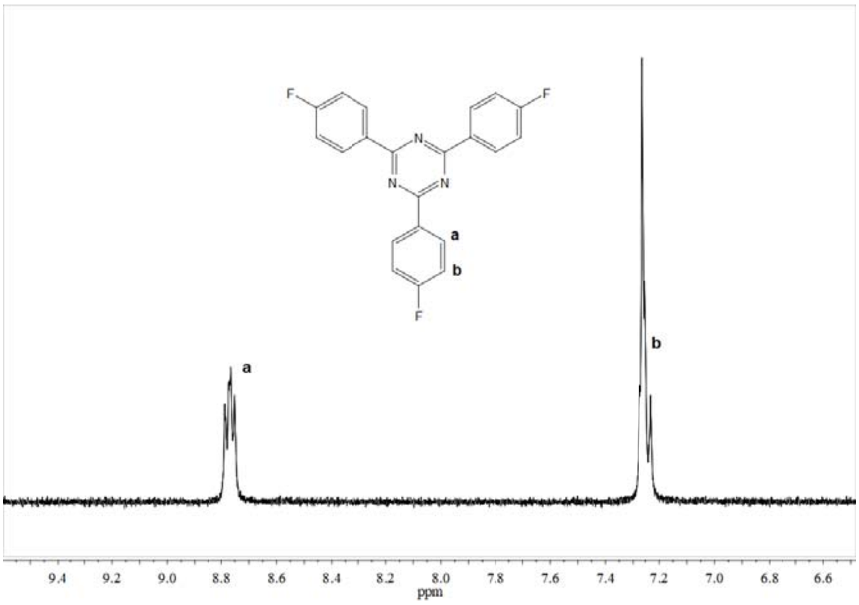

Figure S2.^{1}H NMR spectrum for TFPT.

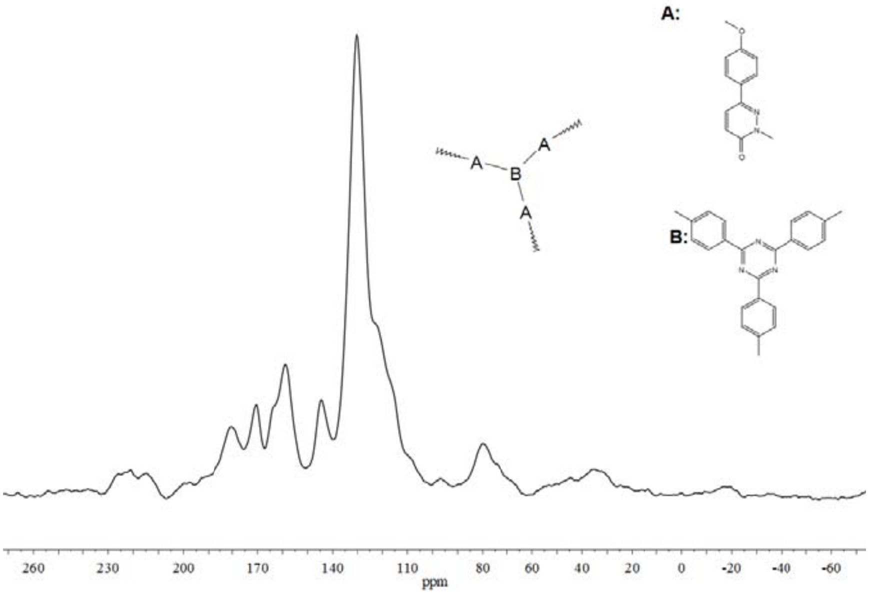

Figure S3.Solid ^{13}C NMR spectrum for the polymer.

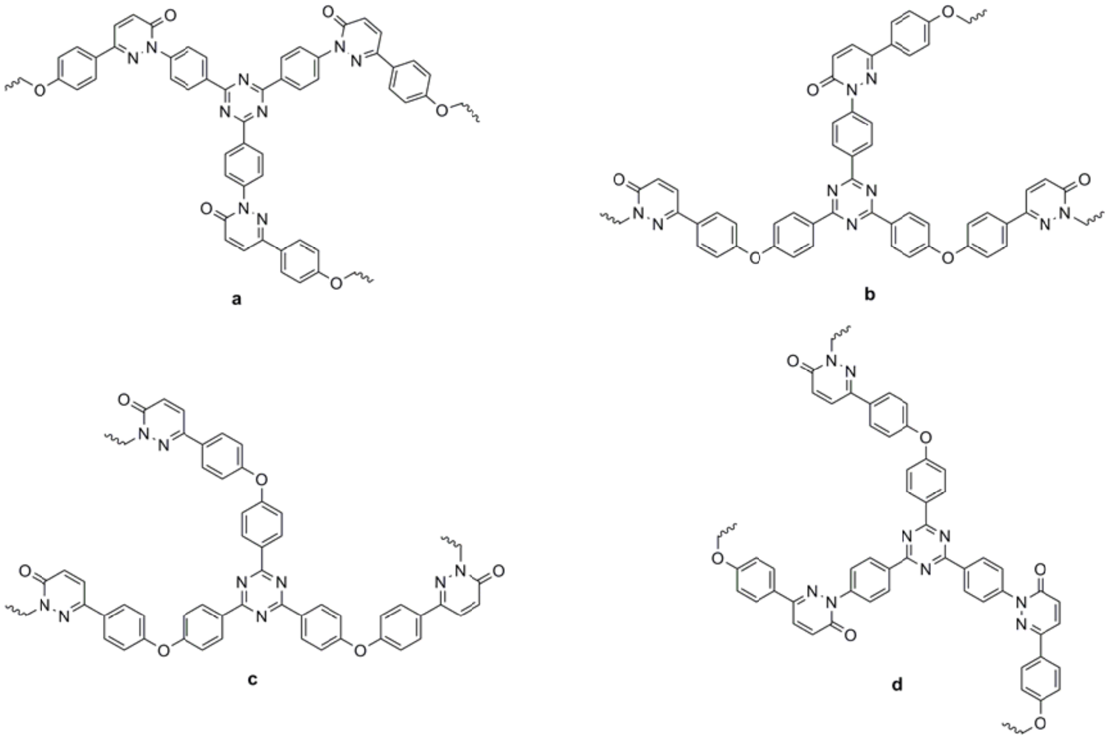

Scheme S1. Chemical formula of the cross-linked polymer.
